# Differential gene expression in recombinant *Pichia pastoris *analysed by heterologous DNA microarray hybridisation

**DOI:** 10.1186/1475-2859-3-17

**Published:** 2004-12-20

**Authors:** Michael Sauer, Paola Branduardi, Brigitte Gasser, Minoska Valli, Michael Maurer, Danilo Porro, Diethard Mattanovich

**Affiliations:** 1 Institute of Applied Microbiology, Department of Biotechnology, University of Natural Resources and Applied Life Sciences, Muthgasse 18, A-1190 Vienna, Austria; 2 Department of Biotechnology and Biosciences, University of Milano-Bicocca, Piazza della Scienza, 2, I-20126 Milan, Italy

## Abstract

**Background:**

*Pichia pastoris *is a well established yeast host for heterologous protein expression, however, the physiological and genetic information about this yeast remains scanty. The lack of a published genome sequence renders DNA arrays unavailable, thereby hampering more global investigations of *P. pastoris *from the beginning. Here, we examine the suitability of *Saccharomyces cerevisiae *DNA microarrays for heterologous hybridisation with *P. pastoris *cDNA.

**Results:**

We could show that it is possible to obtain new and valuable information about transcriptomic regulation in *P. pastoris *by probing *S. cerevisiae *DNA microarrays. The number of positive signals was about 66 % as compared to homologous *S. cerevisiae *hybridisation, and both the signal intensities and gene regulations correlated with high significance between data obtained from *P. pastoris *and *S. cerevisiae *samples. The differential gene expression patterns upon shift from glycerol to methanol as carbon source were investigated in more detail. Downregulation of TCA cycle genes and a decrease of genes related to ribonucleotide and ribosome synthesis were among the major effects identified.

**Conclusions:**

We could successfully demonstrate that heterologous microarray hybridisations allow deep insights into the transcriptomic regulation processes of *P. pastoris*. The observed downregulation of TCA cycle and ribosomal synthesis genes correlates to a significantly lower specific growth rate during the methanol feed phase.

## Background

The methylotrophic yeast *Pichia pastoris *is well established as expression host for heterologous proteins (reviewed by [1] and [2]). However, despite the high technological impact of *P. pastoris*, the physiological and genetic information is still rather scarce. The genome sequence has not been published, and in fact less than 100 complete gene sequences have been deposited with GenBank by the time of writing. Consequently, as for most other non-model species, no DNA microarrays are being manufactured. Hence, one of the most powerful tools for the investigation of changes in expression patterns is not available for this yeast species.

To circumvent this problem, heterologous hybridisation to commercially available DNA microarrays might be conceivable. In fact, the successful non-homologous probing to microarrays has been reported recently. These studies cover a wide variety of organisms, including bacteria [3], a yeast [4], but also plants [5] and metazoan organisms [6-9]. The highest number of cross-hybridisation experiments has been performed with human microarrays. Chismar *et al. *[10] report, for instance, that heterologous probing of human cDNA arrays allows to gain useful information about gene expression in various primates. Moody *et al. *[11] compared, furthermore, the reproducibility of species-specific and cross-species hybridisations by evaluation of microarray hybridisations of porcine and human samples to human cDNA arrays. They reported that results generated by heterologous hybridisation were as reproducible as by homologous hybridisation, and the correlation between data derived from porcine and human hybridisations was strong. As judged from EST sequences of the porcine genome, the authors propose, that stretches of at least 100 bp with high similarity to the human homologue are sufficient for hybridisation. Renn *et al. *[9] compared the performance of cDNA microarrays from an African cichlid fish (*Astatotilapia burtoni*) for the heterologous hybridisation with cDNAs from eight different fish species, some of them closely related (other cichlids) and others more distantly related (among them Atlantic salmon and guppy). They conclude that significant results are obtained even with distantly related species, however, the number of positive spots declines with the phylogenetic distance, as strikingly does the degree of measured regulation.

While yeasts share many morphological and physiological similarities, they represent a very heterogeneous group of fungal organisms, and a high degree of gene sequence similarity cannot be assumed *a priori*. When cDNA of the non-conventional yeast *Zygosaccharomyces rouxii *was probed by cross-hybridisation to *Saccharomyces cerevisiae *GeneFilters, only 155 ORFs out of the *Z. rouxii *genome could be reproducibly detected [4]. Anyhow, 86 genes out of these showed altered expression patterns between non-stressed and salinity-stressed *Z. rouxii *cells and 38 genes behaved differently than the *S. cerevisiae *homologues, indicating that the information gained is limited but nevertheless useful. As judged from 26S ribosomal DNA sequences, *Z. rouxii *is assumed to be more closely related to *S. cerevisiae *than *P. pastoris*, but still, all three belong to the hemiascomycetes [12].

As there are not many genes characterised for *P. pastoris*, there is no simple way to assess the degree of gene sequence similarity between *P. pastoris *and *S. cerevisiae*. However, in many of the few genes sequenced, stretches of high similarity (score >75% over a length of at least 250 bp and more) can be identified. Most of the known genes belong to the carbon and energy metabolism or contribute to amino acid or protein synthesis. Another group of *P. pastoris *genes with known sequence belongs to pathways specific for methylotrophic yeasts. For these genes there are no homologues present in *S. cerevisiae*. Evidently, this respective fraction of the *P. pastoris *genome would remain unevaluated by heterologous hybridisation.

The main aims of this work are to verify whether a heterologous DNA array approach allows to obtain useful information for *P. pastoris*, and to identify genes that are specifically regulated upon a shift from glycerol to methanol as carbon and energy source. This shift is of particular interest since one of the specific features of methylotrophic yeasts is the tightly regulated methanol metabolism, which has been utilised for the construction of strong and tightly controlled expression vectors. The methanol induced promoter of the alcohol oxidase 1 (*AOX1*) gene, which is repressed by many carbon sources such as glucose, glycerol or ethanol, is widely used for heterologous gene expression in *P. pastoris*. Accordingly, methanol is often used as the carbon source that induces the production of heterologous proteins. In addition to heterologous protein induction, the shift of the carbon source to methanol causes major structural and physiological changes within the cell. The enzymes for methanol metabolism are synthesised de novo and some of them are translocated into peroxisomes. Strikingly, peroxisomes can fill most of the cellular volume and AOX1 alone can account for up to 35% of the total soluble protein [13]. Additionally, heterologous protein production and environmental conditions like low fermentation pH have been proven to exert stress in recombinant *P. pastoris *[14]. Hence, for a first study of the transcriptomic regulations of recombinant *P. pastoris*, we used a strain expressing human trypsinogen under control of the *AOX1 *promoter, under conditions that strongly influence the physiology of the host cells, as previously described [15,16]. A series of microarray hybridisations was performed as depicted in table 1, first to qualify the feasibility of cross-species hybridisation, and secondly to analyse the effects of the substrate change in fed-batch fermentations.

## Results and discussion

### 1. Qualification of heterologous hybridisation

Before analysing differential gene expression data, it was our intention to verify whether the heterologous hybridisation of *S. cerevisiae *DNA microarrays with *P. pastoris *cDNA results in significant data. Obviously, the intensity of a signal will depend both on the amount of the specific mRNA in the sample, and the sequence similarity with the respective gene of *S. cerevisiae*. Therefore, we compared the signals obtained from four microarrays hybridised with *P. pastoris *cDNA with four microarrays hybridised with *S. cerevisiae *cDNA (as a control), obtained from shake flask cultures.

To estimate the overall potential to obtain data, and the degree of loss of information, the total number of genes giving significant values, and those determined to be under a given threshold were compared (table 2). In average, 66 % of all genes present on the microarray were either only weakly transcribed, not similar enough to produce a significant signal or not present at all in *P. pastoris*. In contrast, by hybridisation with *S. cerevisiae *cDNA about 46 % of all genes remained undetected.

We analysed those genes of *P. pastoris *for which sequences were deposited in the GeneBank database for sequence similarities to the *S. cerevisiae *genome, and determined the number of significant spots on 6 microarrays. 66 % of the signals derived from genes with high similarity (score > 75 % along stretches longer than at least 250 bp) were significant, while of the moderately to weakly similar genes only 28 % of the signals were significant. This indicates that a high sequence similarity of a sub-sequence within a gene is sufficient for efficient hybridisation. Considering that the signal intensity will depend on sequence similarity and length, but also on mRNA abundance, it becomes obvious, however, that a distinct minimum threshold of similarity cannot be defined.

It was expected that the number of positive spots would be lower for heterologous hybridisation as compared to homologous hybridisation, but the relatively high number of significant values obtained in our experiment is very promising to achieve useful and new information from this technique. Nevertheless, we sought statistical evidence for the biological significance of hybridisation signals obtained with *P. pastoris *cDNA.

**Figure 1:**
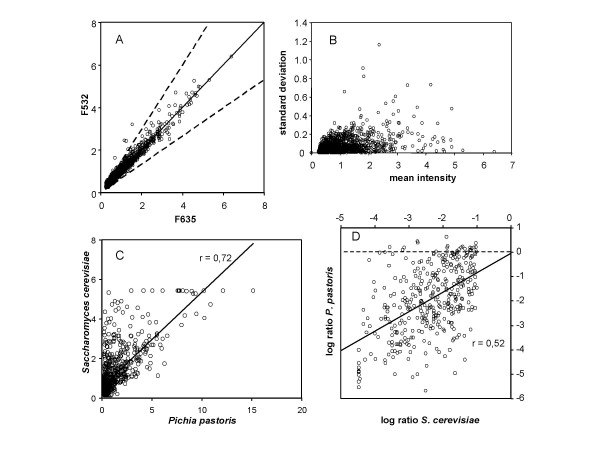
**Statistical evaluations. **(A) "yellow experiment": identical cDNA samples of *P. pastoris *were differently labelled and hybridised to *S. cerevisiae *microarrays. Normalised data of channel 532 nm (Cy3) are plotted against channel 635 nm (Cy5). The solid line represents the linear correlation. Dotted lines indicate the limits of 1.5 fold differences between two signals on one spot. More than 99% of all values vary less than 1.5 fold from each other. (B) Standard deviations of all value pairs as shown in panel A, plotted against the respective mean normalised intensities. (C) Correlation of spot intensities comparing *S. cerevisiae *and *P. pastoris*, grown in identical conditions. (D) Correlation of gene regulation. The correlation between the downregulated genes of *S. cerevisiae *and *P. pastoris *upon a shift from pH 5.0 to pH 3.5 is shown.

First of all, data obtained from microarrays that were hybridised with identical but differentially labelled samples (yellow experiment) were evaluated, showing very high correlation coefficients of 0.97 and 0.98, for *S. cerevisiae *and *P. pastoris *respectively (table 3 and Fig. 1A). The correlation of data from identical samples on different microarrays is somewhat lower (r = 0.86 – 0.92), due to different relative intensities on different chips.

Since usually the data of two samples on one microarray are to be compared, it was important to evaluate the reproducibility, expressed as the standard deviations of both values of each spot on microarrays hybridised with identical samples. Fig. 1B displays the standard deviations plotted against the mean relative intensities of all significant spots of such a *P. pastoris *experiment, showing that the standard deviations do not vary over a wide range of signal intensities, which is in concordance with the results of Moody *et al. *[11]. 96 % of the signals have a relative standard deviation (s. d. divided by mean) below 0.2.

Furthermore, we evaluated the correlation between the signal intensities obtained from *P. pastoris *cDNA with that from *S. cerevisiae *cDNA (cells grown under the same conditions). A highly significant correlation (r = 0.72) was observed (Fig 1C). Considering expectable differences in gene expression, different sequence similarities and the fact that signals from two microarrays were compared, such a high correlation is remarkable and suggests that the data obtained are biologically meaningful. The results obtained are in line with the data published by Moody *et al. *[11] who showed a strong correlation between porcine and human samples on human microarrays. Our data also support Renn *et al. *[9] who demonstrated that both the number of significant spots and signal correlation of different fish samples on *A. burtoni *microarrays depended on the phylogenetic distances between sample species and test species.

To evaluate the significance level for up- and downregulation, the signals from yellow experiments were plotted one against the other (Fig. 1A). These values should obviously fall in the unregulated range. A threshold regulation factor of 1.5 (illustrated by the dotted lines) includes 99.1 % of all significant spots as not significantly different, which means that such a threshold would yield false positives for less than 1 % of the significant genes.

Finally, the global regulation pattern of *P. pastoris *and *S. cerevisiae *between pH 5.0 and 3.5 was compared by correlating all genes downregulated under acidic conditions (Fig. 1D). The correlation is highly significant (p < 0.01) with a correlation coefficient r = 0.51. Clearly, different gene regulation, as well as different degrees of sequence similarities contribute to a reduction of this correlation. Interestingly, the average fold signal change of regulated genes is lower for *P. pastoris *than for *S. cerevisiae *(correlation slope = 0.8). A similar observation was made for more distantly related fish species [9].

### 2. Analysis of gene regulation in *P. pastoris*

**Figure 2:**
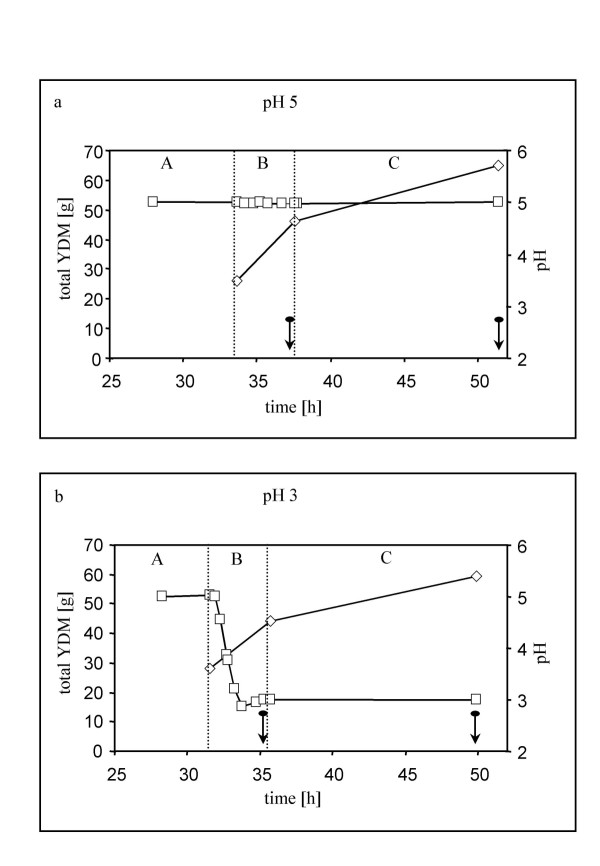
**Fed batch fermentations of *P. pastoris. ***The two panels represent the two different fermentations performed: (a) The pH was kept at 5.0 throughout the fermentation. (b) The pH was let drop to 3.0 and was kept constant subsequently. (A) indicates the batch phase, the cells were growing on glycerol. The time scale starts at 25 h. (B) indicates the glycerol fed batch phase and (C) indicates the methanol fed batch phase. Methanol induces heterologous protein production and serves as a carbon source at the same time. The diamonds (◇) show the total yeast dry mass and refer to the left scale. The squares (□) show the pH of the culture broth and refer to the right scale. The arrows indicate the time points, when the samples for microarray analysis were taken.

As an example for differential gene expression in this study, the level of transcripts during the methanol induction phase of a lab scale fermentation was compared to that during the glycerol feeding phase, both at pH 5.0, and at pH 3.0. Figure 2 shows the development of biomass over time along with the pH and indications of feed changes, and furthermore depicts the time points when the analysed samples were taken.

Of all genes that gave significant signals, we report those that showed significant differences upon the shift from glycerol to methanol under at least one condition (pH), and that have a defined function in *S. cerevisiae*.

Unsurprisingly, genes involved in the core carbon metabolism show a significantly different expression during methanol and glycerol metabolism (table 4). Almost no differences between the samples obtained at pH 5.0 and 3.0 were found. Fructose-1, 6-bisphosphatase was significantly upregulated at least in one sample, whereas fructose 1, 6-bisphosphate aldolase, glyceraldehyde-3-phosphate dehydrogenase, enolase, and pyruvate kinase were found to be downregulated on methanol. Transketolase expression was enhanced while the transaldolase transcript level was relatively reduced. Of these enzymes, fructose-1, 6-bisphosphatase and transketolase (among others) are needed for biomass synthesis on methanol. Some genes related to ATP production were found to be downregulated which appears plausible as the rate of energy consumption is lower at the significantly lower specific growth rate when methanol is the carbon source. The beta subunit of pyruvate dehydrogenase was significantly downregulated at pH 5.0, further indicating that methanol metabolism decreases the TCA cycle flux. The upregulation of pyruvate decarboxylase under at least one condition comes somewhat unexpected, because an increase of the flux to ethanol production appears questionable for cells growing on methanol.

Table 5 displays a list of ribosomal genes that are essentially all downregulated on methanol. Considering the decreased specific growth rate, a decrease of total RNA is generally expected due to a decreased overall protein synthesis. Interestingly, four histone genes that gave significant signals were not regulated at pH 5.0, but induced at pH 3.0.

**Figure 3:**
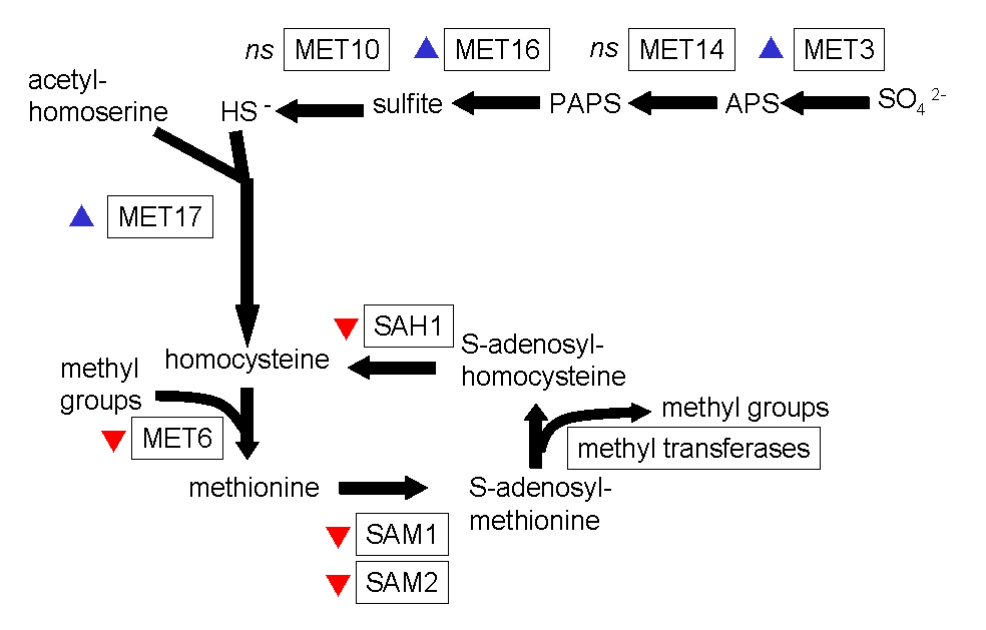
**Schematic representation of the observed regulations in the sulfur, methionine and S-adenosyl-methionine metabolism. **Gene names are framed. APS: adenylylsulfate, PAPS: 3'-phosphoadenylyl-sulfate, *MET3*: ATP sulfurylase, *MET6*: N5-methyltetrahydrofolate homocysteine methyltransferase, *MET10*: sulfite reductase, *MET14*: adenylylsulfat kinase, *MET16*: 3'-phosphoadenylyl-sulfate reductase, *MET17*: O-acetylhomoserine (thiol)-lyase, *SAH1*: S-adenosyl-L-homocysteine hydrolase, *SAM1*: S-adenosylmethionine synthetase, *SAM2*: S-adenosylmethionine synthetase. (▲) upregulation upon shift from glycerol to methanol; (▼) downregulation upon shift from glycerol to methanol; (*ns*) no significant values obtained.

Only a few amino acid biosynthesis genes appeared to be regulated (table 6), indicating that amino acid synthesis is turned on, both on glycerol and methanol, as mineral media were used throughout the experiment. A major exception was the significant regulation of the proteins involved in methionine metabolism. While the homologues to *MET3*, *MET16 *and *MET17 *were upregulated on methanol, the homologues to *MET6*, *SAM1*, *SAM2 *and *SAH1 *were downregulated. As shown in figure 3, the first group of enzymes catalyses the reduction and fixation of sulphur, while the second group drives the cycle responsible for methyl group donation. The reduction of this pathway in cells grown on methanol would imply a decrease of the flux from the C1-pool to methionine by *MET6 *(5-methyltetrahydrofolate-homocysteine S-methyltransferase), which catalyses the transport of activated methyl groups from 5-methyl-THF to homocysteine. These methyl groups are then passed on via S-adenosyl-methionine by a variety of S-adenosyl-L-methionine-dependent methyl transferases, many of them being involved in ribosomal subunit biogenesis, rRNA and tRNA-processing, mRNA capping and nuclear export – once again stressing the lower demand for protein synthesis rate upon growth on methanol.

Other significantly regulated groups of genes (table 7) belong to the thiamine biosynthesis, all being upregulated, and the so-called snooze genes related to the stationary phase. Two of these genes, *SNZ1 *and *SNZ2*, were differently regulated at pH 5 and pH 3, being repressed on methanol at pH 5 and induced at pH 3, while *SNZ3 *was repressed at pH 5, but did not yield a significant value at pH 3. However, the reported high sequence similarity of the *S. cerevisiae SNZ *genes will not enable a reliable differentiation of their regulation on microarrays. For *S. cerevisiae *it is reported that the highly homologous products of the *SNZ *gene family are involved in vitamin B6 (pyridoxal) synthesis [17]. Zeidler *et al. *[18] postulated that pyridoxal is a precursor of thiamine in yeast. Accordingly, the *SNZ *genes have been reported to be induced both by thiamine and pyridoxal depletion [17]. However, with the data obtained in our experiment we cannot interpret the differential behaviour of the *SNZ *and *THI *genes, since among the genes utilising thiamine-pyrophosphat (TPP) as cofactor, transketolase (*TKL1*) and pyruvate decarboxylase (*PDC1*, *PDC5*) are upregulated while *PDB1 *(pyruvate dehydrogenase beta-subunit) and presumably also α-ketoglutarate dehydrogenase (belonging to the TCA cyle) and DHAS (belonging to the methanol utilisation pathway) are downregulated.

Thioredoxin-related genes appeared to be regulated upon shift from glycerol to methanol, too (table 7). Those being downregulated at least in one sample (IMP cyclohydrolase, ribonucleotide reductase, and thioredoxin reductase) are involved in ribonucleotide synthesis, again indicating a decreased demand of RNA precursors. Thioredoxin peroxidase, on the other hand, which is involved in the regulation of cell redox homeostasis and response to oxidative stress by reducing H_2_O_2 _and peroxide radicals, tended to be upregulated. This was expected due to the higher amount of oxidative stress during methanol utilisation.

**Figure 4:**
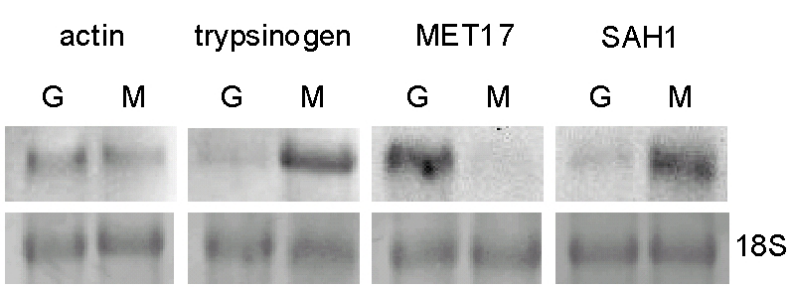
**Northern blot analysis of selected genes. **Exemplarily, the RNA of the pH 5.0 experiment was analysed for the expression of four genes. Actin and human trypsinogen were used as controls for an unregulated, and a methanol induced gene, respectively. *MET17 *(O-acetylhomoserine (thiol)-lyase) and *SAH1 *(S-adenosyl-L-homocysteine hydrolase) were chosen as strongly down- or upregulated genes, as determined before (table 6).

To verify the data obtained with microarrays with an independent method, a northern blot analysis of the total RNA samples from the culture grown at pH 5.0 was performed, using the respective *P. pastoris *homologous sequences to produce the probes (Fig. 4). The actin mRNA level was unchanged whereas the human trypsinogen mRNA level (as a positive control) was strongly induced on methanol, both as expected. *MET17 *and *SAH1 *exemplify differentially regulated genes identified by the microarray experiment (table 6). The data from the northern blot confirmed the results derived by microarray analysis, further underlining the reliability of the method.

Surprisingly, only minor differences in transcriptional regulation were observed between cultures grown at pH 5.0 and pH 3.0. Of course it has to be considered that not the direct effects of a shift in external pH was observed. Still, one could expect a different set of genes to be significantly regulated upon a shift to methanol metabolism at the different pH values. At pH 3.0 a decreased yield in biomass was detected as compared to cultures at pH 5.0, which is consistent with the observation of Hohenblum *et al. *[16] stating that low fermentation pH decreases the viability of *P. pastoris*. In order to compare the general behaviour of *P. pastoris *at different external pH to that of *S. cerevisiae*, we measured the intracellular pH (pH_i_) of the cells. The cells of the culture at pH 5.0 showed a pH_i _of 7.1 at the end of the glycerol phase, and of 7.2 the end of the methanol phase, whereas the pH_i _of both samples of the cultures at pH 3.0 was 7.3. Thus, no changes of the pH_i _were observed between cultures grown at the chosen pH values, which is in contrast to the behaviour of *S. cerevisiae*, where the pH_i _appears to be more dependent on the external pH [19,20].

## Conclusions

We could successfully demonstrate that it is possible to obtain new and valuable information about transcriptomic regulation in *P. pastoris *by probing *S. cerevisiae *DNA microarrays.

Specific regulation upon a shift from glycerol mineral medium to methanol mineral medium under conditions similar to a production process were analysed. As major effects we recognised a downregulation of TCA cycle genes, and a transcriptional decrease of genes related to ribonucleotide and ribosome synthesis. Furthermore, the supply of activated methyl groups via adenosyl methionine was reduced, indicating a decreased ribosome and tRNA synthesis, which is not surprising since the specific growth rate is significantly decreased during the methanol feed phase in comparison to the glycerol feed phase. Correspondingly, a downregulation of the energy metabolism upon methanol induction appears reasonable.

Only a few genes were differentially regulated when comparing expression differences between growth on glycerol and growth on methanol of a culture grown at pH 5.0 to one at pH 3.0. Among the few genes found are the *SNZ *genes and the histone genes, but a plausible hypothesis for the differential pH dependent regulation was not found. Interestingly, also the intracellular pH did not change between the different external conditions, indicating a major difference in pH regulation between *P. pastoris *and *S. cerevisiae*.

## Materials and Methods

Unless stated otherwise, all chemicals were purchased from Merck Eurolab, and all enzymes for DNA manipulation were purchased from MBI Fermentas.

### 1. Strains

The expression strain used in this study was *P. pastoris *strain X33 (Invitrogen), a wild type strain which can grow on minimal media without supplements. The identity of the strain in use was verified by partial 26S ribosomal DNA sequencing (data not shown). The selection mechanism was based on the Zeocin™ resistance of the transformation vector. Transformation of the strain was carried out with a plasmid derived from pPICZαB (Invitrogen), containing the gene for human trypsinogen 1 [21]. pPICZαB utilises the *AOX1 *promoter of *P. pastoris *and the α-factor leader sequence of *S. cerevisiae *for product secretion. The selected strain was of the methanol utilisation positive (mut^+^) phenotype, which means that it is fully capable to metabolise methanol as the sole carbon source.

As a control strain we used *S. cerevisiae *CEN.PK 113-5D (*MATa*, *ura3*) [22].

### 2. Shake flask cultivation of *P. pastoris *and *S. cerevisiae*

Shake flask cultures were performed at 28°C in YPD medium (2% peptone, 2% glucose, 1% yeast extract).

The cells were inoculated to an OD_660 _of 0.3 from a pre-culture grown over night.

For the yellow experiment the samples were taken in exponential phase after 5 h of growth (OD_660 _for *S. cerevisiae*: 0.89, *P. pastoris*: 1.2).

To assess differentially regulated genes the respective cultures were divided after 5 h of growth. One half was incubated as before, whereas the other half was supplemented with 250 mM acetic acid. Samples were collected after shaking for 1.5 h at 28°C. The pH of the untreated culture was 5. The pH of the acid treated culture was 3.5.

### 3. Fermentation of *P. pastoris*

Fed batch fermentations were performed with a MBR mini bioreactor with a final working volume of 2 l, essentially as described by Hohenblum *et al. *[16].

The media were as follows

#### PTM_1 _trace salts stock solution contained per litre

6.0 g CuSO_4• _5H_2_O, 0.08 g NaI, 3.0 g MnSO_4• _H_2_O, 0.2 g Na_2_MoO_4• _2H_2_O, 0.02 g H_3_BO_3_, 0.5 g CoCl_2_, 20.0 g ZnCl_2_, 65.0 g FeSO_4• _7H_2_O, 0.2 g biotin and 5.0 ml H_2_SO_4 _(95 %-98 %). All chemicals for PTM_1 _trace salts stock solution were from Riedel-de Haën, except for biotin (Sigma), and H_2_SO_4 _(Merck Eurolab).

#### Batch medium contained per litre

23.7 ml H_3_PO_4 _(85 %), 0.6 g CaSO_4• _2H_2_O, 9.5 g K_2_SO_4_, 7.8 g MgSO_4• _7H_2_O, 2.6 g KOH, 40 g glycerol, 4.4 ml PTM_1 _trace salts stock solution.

#### Glycerol fed-batch solution contained per litre

632 g glycerol (100 %) and 12 ml PTM_1 _trace salts stock solution

#### Methanol fed-batch solution contained per litre

988 ml methanol (100 %) and 12 ml PTM_1 _trace salts stock solution

The dissolved oxygen was controlled at DO = 30 % with the stirrer speed (600 – 1200 rpm). Aeration rate was 100 l h^-1 ^air, which was supplemented with oxygen (up to 25 %) after the begin of the fed batch. The temperature was 25°C, and the pH was controlled with NH_3 _(25 %).

Before starting the fermentation, the pH of 1.2 l batch medium was set to 5.0 with NH_3 _(25 %). The batch phase of approximately 32 h was followed by a 4 h fed batch with glycerol medium (feed rate 15.6 ml h^-1^), leading to a dry biomass concentration of approximately 40 g l^-1^. Then, the feed with methanol medium was started with a feed rate of 6.4 ml h^-1^. The fermentation was terminated 14 h after the methanol feed start. The pH was 5.0 during batch, and either kept at 5.0 throughout the fermentation, or decreased to 3.0 at the beginning of the glycerol fed batch. The final dry biomass concentration was 51.4 g l^-1 ^at pH 5.0, and 46.7 g l^-1 ^at pH 3.0.

Samples were taken at the end of the glycerol fed batch phase and at the end of the methanol fed batch phase, respectively, as depicted in figure 2.

### 4. mRNA preparation

The cell pellets were re-suspended in 10 × the volume of TRI-reagent (Sigma) and frozen.

The samples were thawed on ice and after addition of acid washed glass beads the cells were homogenised in a Ribolyser (Hybaid Ltd.) for 2 × 20 sec, in between cooling on ice. After addition of chloroform, the samples were centrifuged and the total RNA was precipitated from the aqueous phase adding isopropanol. The pellet was washed 2 × with 70% ethanol, dried and re-suspended in RNAse free water. mRNA was isolated using the MicroPoly(A)Purist mRNA purification Kit (Ambion) according to the manufacturers protocol.

### 5. Synthesis and labelling of cDNA

5 μg of mRNA and 0.5 μg of oligo dT primer were mixed in 7 μl of water, incubated for 5 min at 70°C and subsequently at 42°C for about 3 min. The following components were added to 5 μl of said reaction mixture: 4 μl reaction buffer (5 x) for SuperScript II reverse transcriptase (Invitrogen), 2 μl dTTP (2 mM), 2 μl dATP, dGTP, dCTP (5 mM), 2 μl DTT (100 mM), 2.5 μl RNasin (40 U, Promega) and 2 μl FluoriLink Cy3-dUTP (1 mM) or 2 μl FluoriLink Cy5-dUTP (1 mM, Amersham Biosciences) respectively, and 1 μl SuperScript II reverse transcriptase (200 U, Invitrogen) to result in a total of 19.5 μl. The mixture was incubated for 1 h at 42°C. After addition of further 200 U SuperScript II reverse transcriptase the mixture was incubated for another 1 h at 42°C. 7 μl of 0.5 M NaOH/50 mM EDTA were added and the mixture was incubated at 70°C for 15 min. The reaction mixture was neutralised by addition of 10 μl Tris-HCl pH 7.5 (1 M). The labelled cDNA of the two corresponding samples were pooled and purified with Qiaquick purification columns (Qiagen) according to the manufacturer's protocol.

### 6. Chip hybridisation and set-up of microarrays

The cDNA microarrays used for this study were Hyper Gene Yeast Chips from Hitachi Software Engineering Europe AG. According to the manufacturer, about 0.1 to 0.3 ng of PCR amplified cDNA (approximately 200 bp to 8000 bp) were spotted onto a poly-L-lysine coated glass slide and fixed by baking, succinic anhydride blocking and heat denaturation.

Labelled cDNA was resuspended in about 70 μl of 5 × SSC/0.05% SDS, heat denatured at 95°C for 3 min and cooled on ice. SDS crystals appearing were dissolved by short and slight warming and the mixture was gently applied to a Yeast Chip according to the scheme presented in table 1. The spotted area was covered with a cover glass and the chips were placed in an airtight container with a humidified atmosphere at 60°C for 16 h.

The cover glasses were removed in 2 × SSC/0.1% SDS and the chips were washed consecutively for 5–10 min each in 2 × SSC/0.1% SDS, 0.5 × SSC/0.1% SDS, and 0.2 × SSC/0.1% SDS at RT. The chips were centrifuged at 600 rpm for 3 min in order to dry them. The washing conditions were chosen according to the manufacturer's manual. We have tested less stringent washing conditions which led to higher background without increasing the number of positive signals.

### 7. Data acquisition and processing

Images were scanned at a resolution of 50 μm with a G2565AA Microarray scanner (Agilent) and were imported into the GenePix Pro 4.1 (Axon Instruments) microarray analysis software. GenePix Pro 4.1 was used for the quantification of the spot intensities. Each appearing gene spot was averaged. The data set was then imported into GeneSpring 6.1 (Silicon Genetics) for further normalisation and data analysis.

All of the values of each channel on each chip were divided by their respective median for normalisation. Subsequently, the median intensity of all 84 TE spots (spotted with buffer, no DNA) deduced from each value, and all spot values less then the standard deviation of said 84 threshold values were considered to be not significant and were set to the value of the standard deviation. To determine induction or repression of gene activity, the normalised signals on each spot were compared, and all genes showing a signal difference exceeding the threshold (2 fold for *S. cerevisiae*, and 1.5 fold for *P. pastoris*, see results) on both parallel independent microarrays were judged as significantly regulated.

### 8. Statistical evaluation of microarray data

After normalisation and background deduction, pairwise correlations of all significant values were calculated using Pearson's correlation coefficient. To evaluate the variability of data derived from both dyes on one chip, the standard deviations of all significant spots hybridised with two identical samples were plotted against the respective normalised mean intensity value. To judge the correlation of gene regulation between *S. cerevisiae *and *P. pastoris*, the regulation factors of all of genes that were significantly downregulated in *S. cerevisiae *upon a difference of pH 5.0 to 3.5, were correlated to the respective *P. pastoris *regulation factor upon the same media difference, using the Pearson's correlation coefficient.

The significance of all correlations against randomly distributed values were evaluated by a t-test, applying a significance level p < 0.01. To exclude an effect of data clustering (as the majority of the values are rather low) on correlation, Spearman's correlation coefficients were calculated as well. As these differed only slightly from Pearson's coefficients, they are not shown.

Linear regression analysis was performed with the regulation intensities of *P. pastoris *against *S. cerevisiae *in order to compare the average fold change observed for both yeasts.

### 9. Determination of the intracellular pH (pH_i_)

The pH_i _was determined as described by Valli *et al. *[19]. Essentially, samples were centrifuged and resuspended in McIlvaine buffer [23] at pH 3.0, containing 20 μM carboxy SNARF-4F AM (Molecular Probes). Loaded cells were analysed on a FACS Calibur (Becton Dickinson, Franklin Lakes, NJ USA) with a 488 nm argon-ion laser. 10^4 ^cells were measured per analysis, using PBS as the sheath fluid. Carboxy SNARF-4F fluorescence emission was measured through a 585/21 BP filter (FL2) and a 670 LP filter (FL3). Threshold settings were adjusted so that cell debris were excluded from data acquisition. The ratio of the two fluorescence intensities is a measure for the internal pH. Calibration was performed with amphotericin B (Sigma) perforated cells as described in Valli *et al. *[19].

### 10. Northern blot analysis

Northern blot analysis was essentially performed as described by Sambrook *et al. *[24]. In short, total RNA prepared as described above was fractionated on a denaturing formaldehyde containing gel, capillary blotted onto a nylon membrane (Nytran Supercharge, Schleicher & Schuell) and fixed by baking. The membrane was stained with methylene blue (0.04% in 0.5 M NaOAc, pH 5.2) for quality control and to ensure that equal amounts of RNA had been loaded.

The probes were PCR amplified from genomic *P. pastoris *DNA using the following primers: actin: *gttccagccttctacgtttctattca *and *acggagtactttctttctggtggag*; *SAH*: *agctgaacttgattttggacgac *and *acttgaggcttgatgttgctgac*; *Met17*: *tgcatcaatggtcacggtaaca *and *tggtgagtagagtagtaaggagcaatga*. The probe for human trypsinogen was prepared as described in [15]. The probes were DIG labelled using the PCR DIG Labeling Mix (Roche) according to the manufacturer's protocol.

Pre-hybridisation and hybridisation were performed in high SDS hybridisation buffer at 42°C.

The blots were washed twice at RT with 2 × SSC/0.1 % SDS and two times at 68°C with 0.5 × SSC/0.1 % SDS. Staining of the blots was performed using anti-Digoxigenin-alkaline phosphatase Fab Fragments (Roche) and the CDP Star chemiluminescent reagent (Tropix) according to the manufacturer's protocol. The images were taken with a Lumi imager F1 (Boehringer Mannheim).

## Authors' contributions

MS and PB designed and performed the microarray hybridisation experiments. MS and BG analysed and interpreted the microarray data, and drafted the manuscript. MV performed the pH_i _determinations. MM ran and analysed the fed batch fermentations. DP participated in the design of this study, and in data interpretation. DM participated in the design of this study, and in data interpretation, performed the statistical analyses, and drafted part of the manuscript. All authors read and approved the final manuscript.

## Figures and Tables

**Table 1:** Set-up of microarrays.

Chip No.	Sample labelled with Cy3	Sample labelled with Cy5	Experiment
1	*P. pastoris *shake flask, pH 5.0	*P. pastoris *shake flask, pH 5.0	yellow
2	*S. cerevisiae *shake flask, pH 5.0	*S. cerevisiae *shake flask, pH 5.0	yellow
3	*P. pastoris *shake flask, pH 5.0	*P. pastoris *shake flask, pH 3.5	pH shift
4	*S. cerevisiae *shake flask, pH 3.5	*S. cerevisiae *shake flask, pH 5.0	pH shift
5	*P. pastoris *fed batch, glycerol, pH 5.0	*P. pastoris* fed batch, methanol, pH 5.0	Shift glycerol to methanol at pH 5
6	*P. pastoris *fed batch, methanol, pH 5.0	*P. pastoris *fed batch, glycerol, pH 5.0	Shift glycerol to methanol at pH 5
7	*P. pastoris *fed batch, glycerol, pH 3.0	*P. pastoris *fed batch, methanol, pH 3.0	Shift glycerol to methanol at pH 3
8	*P. pastoris *fed batch, methanol, pH 3.0	*P. pastoris *fed batch, glycerol, pH 3.0	Shift glycerol to methanol at pH 3

The table indicates the labelling of the samples and which samples are hybridised together on one microarray for which experiment. The chip numbering is arbitrary. "Yellow" experiment indicates that identical samples were labelled with both dyes and hybridised to the same microarray to test reproducibility.

**Table 2:** Comparison of the number of significant values obtained from homologous *versus *heterologous microarray hybridisations.

	Significant values	Values under threshold
*P. pastoris*	2031 ± 206	3906 ± 206
*S. cerevisiae*	3086 ± 888	2851 ± 888

Mean values of 4 microarrays each, and their respective standard deviations are shown.

**Table 3:** Pairwise Pearson's correlation coefficients.

Sample 1	Sample 2	microarray	correlation coefficient
*P. p. *shake flask pH 5.0	*P. p. *shake flask pH 5.0	same	0.98
*S. c. *shake flask pH 5.0	*S. c. *shake flask pH 5.0	same	0.97
*P. p. *shake flask pH 5.0	*P. p. *shake flask pH 5.0	different	0.90
*S. c. *shake flask pH 5.0	*S. c. *shake flask pH 5.0	different	0.92
*P. p. *shake flask pH 5.0	*S. c. *shake flask pH 5.0	different	0.72
*P. p. *fed batch, glycerol pH 5.0	*P. p. *fed batch, glycerol pH 5.0	different	0.86
*P. p. *fed batch, methanol pH 5.0	*P. p. *fed batch, methanol pH 5.0	different	0.92
*P. p. *fed batch, glycerol pH 3.0	*P. p. *fed batch, glycerol pH 3.0	different	0.90
*P. p. *fed batch, methanol pH 3.0	*P. p. *fed batch, methanol pH 3.0	different	0.83

Normalised significant signal intensities derived both from the same and from different microarrays were correlated. *P. p *= *P. pastoris*, *S. c. *= *S. cerevisiae*.

**Table 4:** Differentially expressed genes of the core metabolism and ATP synthesis for which significant values were obtained.

Metabolic Pathway	Common Gene Name	pH 5	pH 3	
Core metabolism	CDC19	▼	▼	Pyruvate kinase
	ENO1	▼	▼	Enolase
	FBA1	▼	▼	Fructose 1, 6-bisphosphate adolase
	FBP1	▲	n.s.	Fructose-1, 6-bisphosphatase
	GPM1	—	▼	phosphoglycerat mutase
	PCK1	— tend.	▼	Phosphoenolpyruvat carboxylkinase
	PDB1	▼	▼	Pyruvate dehydrogenase (beta subunit)
	PDC1	▲	n.s.	Pyruvat decarboxylase
	PDC5	▲	?	Pyruvat decarboxylase
	TAL1	▼	▼	Transaldolase
	TDH1	▼	▼	Glyceraldehyde-3-phosphate dehydrogenase
	TDH2	▼	▼	Glyceraldehyde-3-phosphate dehydrogenase
	TDH3	▼	▼	Glyceraldehyde-3-phosphate dehydrogenase
	TKL1	▲	▲	Transketolase 1
	FDH1	▲	▲	protein with similarity to formate dehydrogenase
	FDH2	▲	▲	Formate dehydrogenase
	SFA1	▲	▲	formaldehyde dehydrogenase

ATP synthesis	IDP1	▼	▼	isocitrate dehydrogenase
	IDP2	▼	n.s.	isocitrate dehydrogenase
	YJL045W	▼	n.s.	succinate dehydrogenase (ubiquinone) activity

A 1.5 fold change in expression was regarded as significant difference. (▲) upregulated upon shift from glycerol to methanol, (—) expression unchanged upon shift from glycerol to methanol, (▼) downregulated upon shift from glycerol to methanol, (n.s.) no significant values were obtained, (tend.) signifies that one of the two microarrays did not allow to obtain a significant value, or that one of the values is changed less than 1.5 fold, a question mark (?) signifies that both values for this experiment are significant but show the opposite change of expression, the result is therefore not interpretable.

**Table 5:** Differentially expressed ribosomal and histone genes for which significant values were obtained (Symbols are as for table 4).

Metabolic Pathway	Common Gene Name	pH 5	pH 3	
Ribosomal Genes	RPL2A	▼	▼	
	RPL4B	▼	▼	
	RPL5	▼	▼	
	RPL6A	—	▼	
	RPL9B	—	▼	
	RPL17A	▼	▼	
	RPP0	▼	▼	
	RPS0A	▼	▼	
	RPS0B	▼	▼	
	RPS1A	▼	▼	
	RPS4B	▼	▼	
	RPS9B	▼	▼	
	RPS12	▼	▼	
	RPS13	▼	▼	
	RPS22A	▼	▼	
	RPS26B	▼	▼	

Histones	HHF1	—	▼	Histone H4
	HHF2	—	▼	Histone H4
	HTB1	—	▼	Histone H2B
	HTA1	—	▼	Histone H2A

**Table 6:** Differentially expressed genes of the biosynthetic pathways of methionine and other amino acids for which significant values were obtained (Symbols are as for table 4)

Metabolic Pathway	Common Gene Name	pH 5	pH 3	
Methionine	MET3	▲	▲	ATP sulfurylase
	MET16	▲	▲ tend.	3'-phosphoadenylsulfate reductase
	MET17	▲	▲	O-acetyl homoserine-O-acetyl serine sulfhydrylase
	MET6	▼	▼	methionine synthase
	SAM1	▼	—	S-adenosylmethionine synthetase
	SAM2	▼	▼	S-adenosylmethionine synthetase
	SAH1	▼	▼	putative S-adenosyl-L-homocysteine hydrolase

Amino acid biosynth.	AAT2	▼	▲	aspartate transaminase
	ARG1	—	▼	arginine biosynthesis
	ILV5	—	▼	branched-chain amino acid biosynthesis
	LPD1	▼	—	serine biosynthesis

**Table 7:** Differentially expressed thiamine biosynthetic pathway genes, stationary phase, and thioredoxin related genes for which significant values were obtained (Symbols are as for table 4)

Metabolic Pathway	Common Gene Name	pH 3	pH 5	
Thiamine biosynthesis	THI4	▲	▲	protein required for thiamine biosynthesis
	THI5	▲	▲	proteins involved in synthesis of the thiamine
	THI11	▲	▲	precursor hydroxymethylpyrimidine (HMP);
	THI12	▲	▲	members of a subtelomeric gene family
	THI13	▲	▲	including THI5, THI11, THI12, and THI13

Stationary phase	SNZ1	▼	▲	stationary phase-induced gene
	SNZ2	▼	▲	stationary phase-induced gene
	SNZ3	▼	n.s.	stationary phase-induced gene

Thioredoxin	ADE16	▼ tend.	n.s.	5-aminoimidazole-4-carboxamide ribonucleotide(AICAR) transformylase/IMP cyclohydrolase
	RNR2	▼	▼	Ribonucleotide reductase
	TRR1	▼ tend.	?	Thioredoxin reductase
	TSA1	?	▲	Thioredoxin peroxidase
	TSA2	▲ tend.	▲	Thioredoxin peroxidase
